# Consistency between clinician and patient perspectives on ARV treatment commencement and switching examined

**DOI:** 10.1186/1742-4690-9-S1-P64

**Published:** 2012-05-25

**Authors:** Jeffrey Grierson, Marian Pitts, Rachel Koelmeyer

**Affiliations:** 1 la Trobe University, Melbourne, Australia

## Introduction

In the context of multiple HAART treatment modalities it is critical that common understandings regarding the motivators and barriers to treatment commencement and switching are shared by both prescribing clinicians and PLHIV.

## Materials and methods

We conducted an online survey of 254 people living with HIV (PLHIV) in Australia and structured interviews with 18 clinicians (HIV S100 prescribers). PLHIV had a median age of 47.5 years. Overall, 87.4% of respondents were currently taking ARV; 5.5% had taken ARV in the past but not currently, and 7.1% had never taken ARV. Clinicians were a mix of high caseload experienced practitioners and newer low caseload clinicians.

## Results

When we examined the motivations for and barriers to treatment commencement, PLHIV identified concerns about potential side effects and the psychological consequences of acknowledging potential physical decline. Clinicians were most likely to assess readiness in terms of ability to maintain adherence. PLHIV relied on the clinician initiating and advising treatment commencement. Clinicians identified process of clinical assessment, ARV education and adherence training as the antecedents of treatment initiation.

Similarly with switching treatments, PLHIV identified concerns about side effect profiles, clinical markers and the potential limiting of treatment options. Clinicians identified resistance patterns, regimen potency and adherence concerns as primary motivators for treatment change. PLHIV generally expected any discussion of switching to be initiated by the clinician. Clinicians discussed the process in terms of resistance testing and patient education around treatment options.

Both PLHIV and clinicians identified the relationship between them as the critical component of the processes of commencing and changing ARV treatment. Clinicians were keen to involve PLHIV in the decision-making process and PLHIV had a high degree of trust in their clinicians’ knowledge and judgement.

## Conclusions

Treatment discussions need to occur frequently. There is evidence of resistance on the part of patients to change unless they are experiencing significant side effects. Clinicians may focus unduly on adherence while PLHIV are more concerned about side effects and disease progression. The valued relationship between clinician and PLHIV provides a fertile basis for improved treatment discussion.

